# Host PTX3 Protein and Bacterial Capsule Coordinately Regulate the Inflammatory Response during *Streptococcus suis* Infection

**DOI:** 10.3390/vetsci10030239

**Published:** 2023-03-22

**Authors:** Qiankun Bai, Ruhui Fan, Ningyuan Zhong, Jianan Liu, Xinming Pan, Huochun Yao, Jiale Ma

**Affiliations:** 1 MOE Joint International Research Laboratory of Animal Health and Food Safety, College of Veterinary Medicine, Nanjing Agricultural University, Nanjing 210095, China; 2 Key Laboratory of Animal Bacteriology, Ministry of Agriculture, Nanjing 210095, China; 3 OIE Reference Laboratory for Swine Streptococcosis, Nanjing 210095, China

**Keywords:** Pentraxin 3, inflammatory response, *Streptococcus suis*, capsule, mouse air pouch model

## Abstract

**Simple Summary:**

*Streptococcus suis* serotype 2 (SS2) is a noteworthy zoonotic pathogen that has been responsible for substantial economic losses to the swine industry and a great threat to human health. In this study, the role of Pentraxin 3 (PTX3) during SS2 infection is explored to verify its potential as a new and effective biological agent that could partially resolve the problems posed by SS2. The study’s results showed that PTX3 facilitated the phagocytosis of macrophage Ana-1 against SS2 strain HA9801, and contributed to inflammatory cell recruitment and cytokine IL-6 release in the mouse air pouch; thus, it was conductive to bacterial clearance during the SS2 infection. The presence of capsular polysaccharide of SS2 (CPS2) was identified as being required for PTX3 to trigger the inflammatory activation; this coordinating modulation of the host’s innate immune response during the SS2 infection further suggested that PTX3 is a potential biological agent for the prevention and treatment of streptococcosis caused by SS2. However, the recommended dose of PTX3 must be further evaluated to avoid an excessive inflammatory response.

**Abstract:**

*Streptococcus suis* serotype 2 (SS2) is a noteworthy zoonotic pathogen that has been responsible for large economic losses in pig production and a great threat to human health. Pentraxin 3 (PTX3) is an essential regulator of the innate immune response to bacterial pathogens; however, its role during SS2 infection is not fully understood. In this study, we found that the SS2 strain HA9801 induced a significant inflammatory response in the mouse air pouch model; this response was amplified by the treatment of exogenous PTX3 simultaneously in terms of the results of inflammatory cell recruitment and proinflammatory cytokine IL-6 production. In addition, PTX3 facilitated the phagocytosis of macrophage Ana-1 against SS2 strain HA9801. The supplementation of exogenous PTX3 significantly reduced the bacterial loads in a dose-dependent manner in lungs, livers and bloods of SS2-infected mice compared to the samples with HA9801 infection alone; this finding indicated that PTX3 may facilitate the bacterial clearance through enhancing the host inflammatory response during SS2 infection. Both PTX3 and SS2 capsular polysaccharide (CPS2) were required for the robust inflammatory response, implying that the host PTX3 protein and SS2 surface CPS2 modulate the host innate immune response in concert. All of these results suggested that PTX3 is a potential novel biological agent for the SS2 infection; however, the recommended dose of PTX3 must be evaluated strictly to avoid inducing an excessive inflammatory response that can cause serious tissue injury and animal death.

## 1. Introduction

*Streptococcus suis* serotype 2 (SS2) is a significant zoonotic pathogen causing various swine diseases; the human cases related to this pathogen are often reported as having streptococcal toxic shock syndromes [1]. In 1998 and 2005, two huge outbreaks of human infection caused by SS2 were reported in China, causing 14 and 38 deaths, respectively [2]. To date, SS2 has been found to show complex genetic diversity and diverse phenotypes during infection [3,4], which are responsible for substantially threatening both economic burdens for the swine industry worldwide and human health. In the last few decades, antibiotics have primarily been used to treat streptococcosis caused by SS2 on most large-scale pig farms. The widespread use of antibiotics has led to the appearance of a series of related problems, such as antibiotic residues, food safety issues, etc. Given current efforts to reduce antibiotic use in animals, developing new effective vaccine candidates and therapeutic agents is an urgent priority.

As capsular polysaccharide (CPS) is a critical factor for *S. suis* virulence and a core antigen for *S. suis* serotyping (such as the CPS2 from SS2 strains), it was proposed as a potential vaccine candidate to universally protect against all strains within the same serotype [5]. In SS2, CPS has been verified to play a vital role in anti-phagocytosis [6,7]. Comparing with the wild-type strain, the infection of CPS2 mutants could not cause significant increases in the fever and leukocyte indexes in a pig infection model [6], suggesting that CPS2 may be involved in the inflammatory response induced by the SS2 infection. However, studies have proven that CPS is a poor immunogenic molecule, and there is a very low induction of CPS-specific antibody titers either by repeated injections of purified CPS or vaccination with *S. suis* type 2 bacteria [8,9,10]. Therefore, overcoming its poor immunogenicity is critical for CPS to be an effective vaccine candidate. Furthermore, Pentraxin 3 (PTX3), also called TNF stimulated gene 14 (TSG14), belongs to the pentraxin superfamily [11] and is highly conserved among humans and other species (human and murine proteins share a 92% sequence identity). PTX3 protein is produced by various cells, especially mononuclear phagocytes and endothelial cells [12], and it has the potential to mediate the enlargement of inflammation and innate immunity in tissues [13]. PTX3 has been identified as a soluble pattern recognition receptor [14], which binds to the complement component C1q [15] and several microorganisms, such as fungi [16], bacteria and viruses [17], orchestrating complement activation to resist the selected pathogens [18]. Otherwise, PTX3 facilitates pathogen recognition by macrophages and enhances their phagocytic vitality [19]; it also modulates leukocyte recruitment in inflammation [20], regulating the clearance of the invasive pathogens. PTX3 also has an important regulatory capacity for the progression and outcome of diverse diseases, including COVID-19 [21] and emphysema [22]; thus, it could be used as a biomarker of inflammatory response levels. As a result, PTX3 is a potential therapeutic agent or immunologic adjuvant against various pathogens. Thus, we speculated whether PTX3 is related to CPS2 in the process of resisting SS2 infection.

In this study, we attempted to further characterize how PTX3 interacts with SS2 in the mouse air pouch and systemic infection models. The phagocytic activity of the mouse peritoneal macrophage Ana-1, the inflammatory response of mice stimulated by SS2 strain or CPS2 extraction and the challenge test of SS2 strain HA9801, were used to investigate the effects of PTX3. All of the findings showed that PTX3 coordinately regulated the inflammatory response in the mouse systemic infection model with CPS2, making it a viable biological agent for the prevention and treatment of streptococcosis caused by SS2. However, the recommended dose of PTX3 must be evaluated strictly to avoid triggering an excessive inflammatory response that can cause serious tissue injury and animal death.

## 2. Materials and Methods

### 2.1. Ethics Statement

SPF (specific-pathogen-free) BALB/c mice (five-week-old females) were purchased from Yangzhou University (Comparative Medicine Center, Yangzhou, China). All animal studies were approved by the Experimental Animal Welfare and Ethics Committee of Nanjing Agricultural University, Nanjing, China (Approval ID: SYXK(SU)2021-0086), and performed according to Animal Welfare Agency Guidelines.

### 2.2. Bacterial Strains and Cells

The *S. suis* type 2 strain HA9801 was isolated from a pig with septicemia in our laboratory in Jiangsu province, China. The strain was cultured at 37 °C, with 5% CO_2_ in Todd-Hewitt broth (THB; Becton-Dickinson, Franklin Lakes, NJ, USA) or THB agar (THA). The mouse monocyte macrophage cell line Ana-1 was purchased from a private company (AmyJet Scientific, Wuhan, China), while the mouse macrophage cell line RAW264.7 (ATCC^®^ TIB-71™) and the human laryngeal carcinoma cell line HEp-2 (ATCC^®^ CCL-23™) were preserved in our lab. All of these cells were cultivated at 37 °C, containing 5% CO_2_ in 1640 medium (Gibco, Thermo, Wilmington, NC, USA) with 10% FBS (fetal bovine serum, Gibco). The Chinese hamster ovary cell line CHO-K1 (ATCC^®^ CCL-61™) was preserved in our lab. It was grown in F-12K Nutrient Mixture Kaighn’s Modification (Gibco-Invitrogen, Carlsbad, CA, USA) containing 10% heat-inactivated fetal calf serum (Gibco-Invitrogen, Carlsbad, CA, USA), at 37 °C, in a humidified atmosphere containing 5% CO_2_.

### 2.3. RNA Isolation and qRT-PCR Analysis

To compare the transcriptional level of the PTX3 gene in blood or lung tissue of mice, RAW264.7 cells and HEp-2 were infected with or without strain HA9801. Three five-week-old BALB/c mice were challenged with the strain HA9801 at a dose of 3 × 10^8^ CFU/mouse; after 6 h, the lung and blood were collected for RNA isolation. Furthermore, for the lungs and bloods of three uninfected mice, HEp-2 cells and RAW264.7 cells were also collected. Total RNA was extracted using TRIzol reagent (Vazyme, China), according to the manufacturer’s instructions. The cDNA was then synthesized with the HiScriptII first-strand cDNA synthesis kit (Vazyme) and residual genomic DNA was removed with DNase I (TaKaRa). The relative expression of PTX3 gene was normalized to the housekeeping gene gapdh transcription. The relative fold change was calculated by the threshold cycle (2^−ΔΔCT^) method [23]. The reported values represented the mean ± SD of three independent RNA extractions.

### 2.4. Production, Purification, and Identification of Recombinant Murine PTX3

Full-length cDNA encoding PTX3 of BALB/c mouse was cloned into pIRES2-egfp (Clontech Laboratories, Mountain View, CA, USA) and transfected into CHO-k1 cells (ATCC CCL-61) by Lipofectamine TM 2000 (Life Technologies, Carlsbad, CA, USA). After growing in adhesion for 4 h, hCHO-k1 cells were incubated in CD-CHO (Gibco-Invitrogen, Carlsbad, CA, USA) without fetal calf serum. The conditioned medium was then collected from confluent monolayers after incubation of 48 h [24].

### 2.5. Preparation of Capsular Polysaccharide from S. suis 2

Capsular polysaccharide was prepared as previously described [25,26]. Bacterial strain HA9801 was incubated to logarithmic phase (OD_600_ = 1.0); it was then centrifuged at 3000× *g* rpm for 10 min. The pellets were washed three times with PBS (pH 7.4), sonicated for a 10 s pulse and centrifuged at 12,000× *g* rpm for 10 min; the supernatant was then collected. Cetyltrimethyl ammonium bromide (CTAB) was added to the supernatant to the final concentration of 1%. The compound was mixing sufficiently to produce the precipitate. Subsequently, CaCl_2_ was added to the precipitate to dissociate polysaccharides from CATB, and the supernatant was collected. Isopropanol was added to the supernatant, which stood overnight at 4 °C. The supernatant was collected and added to precooled ethanol to a final concentration of 80%. After being thoroughly shaken, the precipitated polysaccharide was collected by centrifugation and washed twice with anhydrous ethanol and acetone. This polysaccharide precipitate then became the crude CPS extracts of strain HA9801.

### 2.6. Regulating Phagocytosis of S. suis 2 HA9801 by Ana-1

The HA9801 bacterial cells of log phase were washed twice with phosphate buffer solution (PBS, pH 7.4) before being resuspended and adjusted to a concentration of 6 × 10^8^ CFU/mL. 500 μL of the suspension was preincubated at 37 °C for 30 min with different doses of PTX3 (5 μg, 10μg, 20 μg). The mixture of PTX3 and HA9801 cells was then incubated with a 500 μL volume of Ana-1 (6 × 10^6^ cells/mL) cells under the same conditions. The mixture was washed three times with sterile PBS (pH 7.4), fixed with cold absolute methanol, and stained with Giemsa (Sigma Chemical Co., St. Louis, MO, USA) after 1 h of incubation. Experiments were done in triplicate, and in each one, 200 cells were analyzed for each parameter in at least five different fields [27]. The calculation of phagocytosis is made up of two components: phagocytic ability (PA), which is the percentage of Ana-1 cells that have trapped bacterial cells of strain HA9801, and; phagocytic index (PI), which is the average quantity of bacterial cells swallowed by each Ana-1 [28].

### 2.7. Mouse Air Pouch Model

To simulate the environment in vivo, a mouse air pouch model was established in a previous study [28]. Firstly, 5 mL sterilized air was injected subcutaneously into the backs of the mice, followed 3 days later by a second injection of 3 mL of air. By the sixth day, the air pouch had basically formed.

### 2.8. Inflammatory Cells and IL-6 Detection

After 5 h of the indicated treatments, mice were sacrificed, and air pouches were washed with 2 mL of ice-cold saline. The lavage fluid was cooled on ice; the cells were then recovered and counted by the manufacturer. Supernatants were harvested and stored at −80 °C for further cytokine IL-6 detection using specific ELISAs (BD Pharmingen and R&D Systems, San Jose, USA) [28]. Standard curves for the cytokines were obtained using the reference concentrations supplied. Our results are the mean of three independent experiments.

### 2.9. Mouse Infection Assays

Mouse infection assays were performed as previously reported [29]. Mice were intraperitoneally injected with HA9801 3 × 10^8^ CFU/mouse with or without PTX3 (5 μg, 10 μg and 20 μg, respectively). Every 2 h, three infected mice were sacrificed by cervical dislocation and their blood, lungs and livers were harvested, weighed and homogenized in PBS. Subsequently, the homogenates and bloods were serially 10-fold diluted and plated on THA medium to enumerate CFU. At the same time, the cytokine IL-6 in the blood of infected mice was detected by specific ELISAs (BD Pharmingen and R&D Systems).

### 2.10. Statistical Analysis

The Log-rank (Mantel–Cox) test was used to analyze the survival curve in the animal experiments. A two-tailed unpaired *t* test was used for the rest of the experiments. For all tests, a *p* value < 0.05 was considered statistically significant (* *p* < 0.05, ** *p* < 0.01, *** *p* < 0.001, **** *p* < 0.0001). The data reported were pooled from three to five experiments and expressed as mean ± SEM.

## 3. Results

### 3.1. PTX3 Protein Is Responsible for the Significant Inflammatory Response during SS Infection

**Figure 1:**
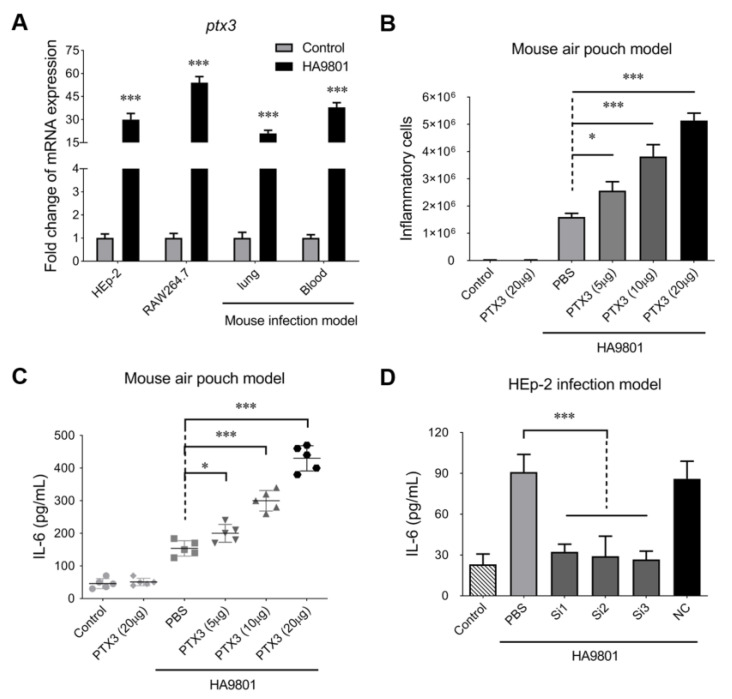
PTX3 contributed to the host inflammatory response during the infection of SS2 strain HA9801. (**A**) the expression of PTX3 in the indicated host cells and organs infected with strain HA9801. The treatments of equal volume PBS were used as control. (**B**,**C**) The recruitment of inflammatory cells (including neutrophils and monocytes identified by the microscopic observation) and release of cytokine IL-6 in the mouse air pouch model infected by strain HA9801 (3 × 10^8^ CFU/mouse) mixed with various doses of PTX3 protein. (**D**) HEp-2 cells were transfected with PTX3 siRNA (Si1-3) or negative control siRNA (NC), and then infected with SS strain HA9801 for 24 h to detect the levels of cytokine IL-6. Statistical significance was determined by a two-tailed unpaired Student’s *t*-test based on comparisons with the PBS group (* *p* <0.05; *** *p* < 0.001).

Our previous studies have identified that the PTX3 protein is involved in the infection of *S. suis* [30,31], though its underlying roles are still not fully understood. In this study, we found that the transcription of gene PTX3 was significantly upregulated (more than a 30-fold change) by the infection of SS2 strain HA9801 on Raw264.7 and Hep2 cells (Figure 1A). In the mouse systemic infection model, the challenge of strain HA9801 caused a huge transcriptional activation of PTX3 in the blood and lungs. These results indicated that host cells recruit and deploy a mass of PTX3 proteins during SS2 infection, which may play vital roles in resisting the invasion by bacterial pathogens. Indeed, PTX3 has been identified as a part of the innate immune system and is one of the acute-phase proteins that perform various functions, such as modulating inflammation, repairing tissue and recruiting immune cells [32]. To investigate the role of PTX3 in the innate immune response during the SS2 infection, a mouse air pouch model was established that assessed the changes in inflammatory levels. As shown in Figure 1B, the supplements of exogenous PTX3 caused significant increases in inflammatory cells in a dose-dependent manner during the infection with SS2 strain HA9801. Similar activations mediated by the exogenous PTX3 were also observed on the IL-6 levels that the air pouch exudates after 5 h of HA9801 infection (Figure 1C). However, neither the supplements of PTX3 nor the HA9801 infection alone could not cause the above phenotypes, suggesting that the inflammatory response may be coordinately regulated by the PTX3 and bacterial infection. To further verify the role of PTX3 in inflammatory modification, we silenced the native PTX3 expression with the specific siRNAs on the Hep2 cells. The results of IL-6 levels showed that knockdown of PTX3 almost completely abolished the activation of the inflammatory response induced by the HA9801 infection (Figure 1D), compared with the cells transfected with the non-specific siRNA (SiNC) and incubated with the PBS control. Overall, our data indicated that PTX3 protein significantly stimulates the host inflammatory response during *S. suis* infection.

### 3.2. The CPS2 Is Required for Triggering the Host Inflammatory Response during SS2 Infection with or without Exogenous PTX3

**Figure 2:**
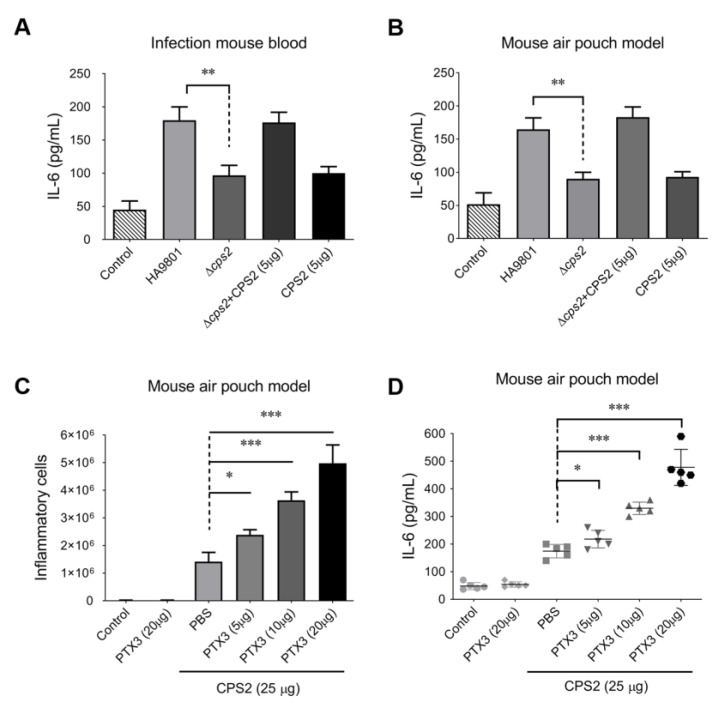
PTX3 facilitated CPS2 to trigger the host’s inflammatory response with or without SS infection. CPS2 contributed to the inflammatory response during SS infection in the mouse systemic infection model (**A**) and mouse air pouch model (**B**). The recruitment of the inflammatory cells (**C**) and release of cytokine IL-6 (**D**) were activated by the co-treatment of CPS2 (25 μg) and PTX3 protein (5, 10, or 20 μg) in the mouse air pouch model. The results represented the mean of three independent experiments. The statistical significance was calculated by two-tailed unpaired Student’s *t*-tests (* *p* < 0.05; ** *p* < 0.01; *** *p* < 0.001).

The capsular polysaccharide of *Streptococcus* species is the dominant surface structure of the organism and plays a critical role in virulence [33]. In this study, the CPS2E gene was deleted under the background of SS2 strain HA9801 to construct a CPS-deficient mutant; this mutant was then designated as strain ΔCPS2. Compared with the wild-type strain HA9801, the deletion of CPS2 significantly attenuated the release of IL-6 in the bloods of infected mice (Figure 2A), while the supplement of 5μg CPS2 could completely recover the attenuation caused by the CPS2 deficiency in strain ΔCCPS2. The injection of 5 μg CPS2 extract into mouse blood alone could not activate the IL-6 release to a similar level as the ΔCPS2 and CPS2 (5 μg) co-treated group. Similar results were observed in the mouse air pouch model (Figure 2B). These observations implied that CPS2 plays an important role in triggering the host’s inflammatory response during the SS2 infection. We then wondered if the additional supplement of CPS2 extraction co-incubated with PTX3 could trigger an inflammatory response in hosts without SS2 infection. As shown in Figure 2C,D, the supplement of 25 μg CPS2 extraction in mouse air pouches treated with different doses of PTX3 protein (5 μg, 10 μg, and 20 μg) could significantly increase inflammatory cells and IL-6 levels. To strengthen the results, the IL-8 levels were also tested in these assays, and showed similar results to the IL-6 levels (Supplementary Figure S1). These findings suggested that the host PTX3 protein and the SS2 surface CPS2 modulate the host inflammatory response in concert.

### 3.3. PTX3 Enhances the Phagocytosis of Ana-1 cells to SS2 Strain HA9801

**Figure 3:**
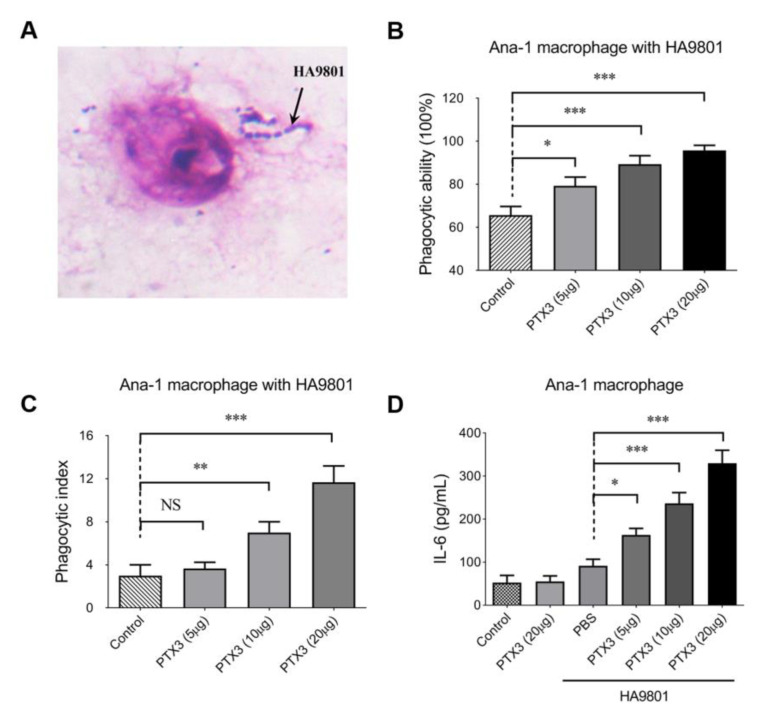
PTX3 enhanced the phagocytosis of mouse macrophage Ana-1 in strain HA9801. (**A**) The observation of Ana-1 swallowing HA9801 stained with Giemsa by microscope (10 × 100). The phagocytic ability (**B**) and phagocytic index (**C**) of macrophage Ana-1 to SS2 strain HA9801 were assessed under the treatment of different doses of PTX3 protein (5, 10, 20 μg). Phagocytic ability was defined as the percentage of Ana-1 cells that trapped bacterial cells of strain HA9801; phagocytic index was defined as the average quantity of bacterial cells swallowed by each Ana-1. (**D**) The levels of the cytokine IL-6 were detected under the treatment of PTX3 protein (5, 10 and 20 μg) during the HA9801 infection. Data were shown as the mean ± SEM of three independent experiments performed in triplicate. Statistical significance was determined by a Student’s *t*-test based on comparisons with the PBS group (ns, *p* > 0.05; * *p* < 0.05; ** *p* < 0.01; *** *p* < 0.001).

As shown in Figure 3A, microscopic observations of methanol-fixed and Giemsa-stained samples confirmed that macrophage Ana-1 has the capacity to trap the HA9801 cells, which were clearly visible and countable. Then, the phagocytic ability of Ana-1 cells was evaluated by calculating the percentage of Ana-1 cells that trapped bacterial cells of strain HA9801. Our data showed that the pretreatment of exogenous PTX3 significantly enhanced the phagocytic ability of Ana-1 cells towards SS2 strain HA9801 in a dose-dependent manner (Figure 3B). Furthermore, the average quantity of bacterial cells swallowed by macrophage Ana-1 was designated as the phagocytic index, which also showed a significantly enhanced effect in the groups pretreated with exogenous PTX3 (Figure 3C). Expectedly, the IL-6 levels of Ana-1 cells pretreated by exogenous PTX3 were remarkably upregulated dose-dependently (Figure 3D). Collectively, all these data suggest that PTX3 facilitates the phagocytic ability of macrophage Ana-1 in SS2 strain HA9801.

### 3.4. PTX3 Contributes to Attenuate the Bacterial Colonization/Proliferation in Multiple Organs during the HA9801 Infection

**Figure 4:**
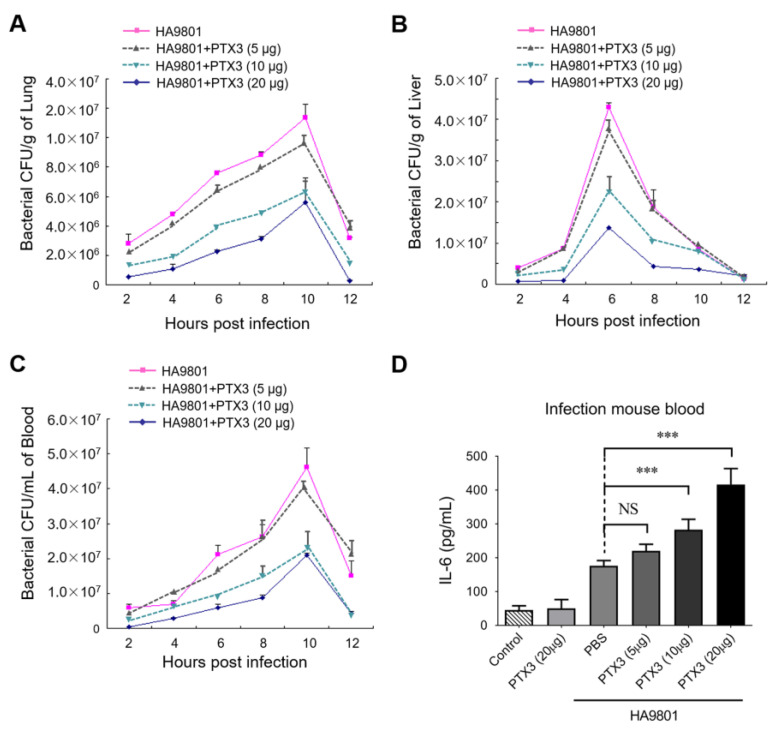
PTX3 significantly reduced the bacterial load in the organs during the HA9801 infection. Bacterial loads in mouse lung (**A**), liver (**B**), and blood (**C**) were detected from 2 to 12 h post-infection, with or without the co-treatment of PTX3 (5, 10 or 20 μg). (**D**) The levels of IL-6 in the HA9801-infected bloods with the co-treatment of PTX3 (5, 10 or 20 μg). Data were shown as the mean ± SEM of three independent experiments performed in triplicate. Statistical significance was determined by a Student’s *t*-test based on comparisons with the PBS group (*** *p* < 0.001).

Next, we explored whether PTX3 plays a vital role in resisting bacterial colonization and proliferation in a mouse systemic infection model (challenged by strain HA9801 at a dose of 2 × 10^8^ CFU/mouse). As presented in Figure 4A–C, the supplement of exogenous PTX3 significantly reduced the bacterial loads in a dose-dependent manner in the lungs, livers, and blood of infected mice compared to the samples from the mice infected with HA9801 alone. The effect was most significant when 20 μg of PTX3 protein was added, which showed the lowest bacterial loads (downregulated at least 3-fold) in the tissues. In addition, IL-6 levels in infected mouse blood showed significant increases in the PTX3-treated groups in a dose-dependent manner (Figure 4D). Altogether, our data indicated that PTX3 may facilitate bacterial clearance through enhancement of the host inflammatory response during SS2 infection.

### 3.5. Intravenous Injection of PTX3 at an Appropriate Dose Significantly Improves Mouse Survival after SS2 Challenge

**Figure 5:**
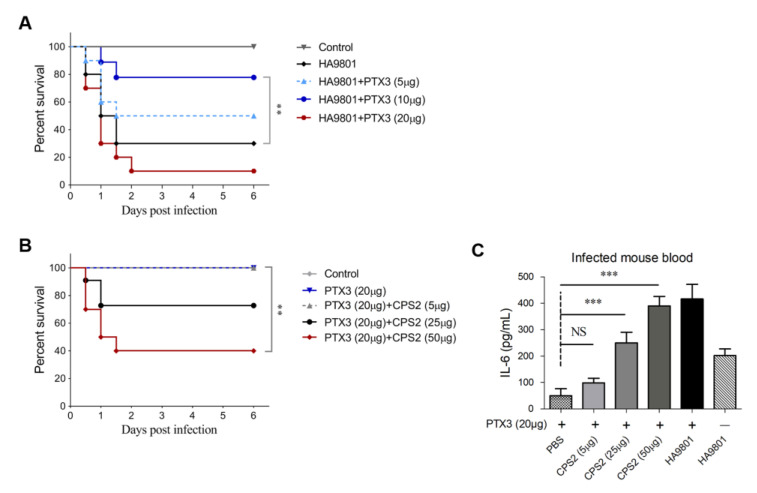
Challenge the protections of PTX3 and CPS2 in mice. (**A**) Survival curves of mice infected by the HA9801 strain with the co-treatment of PTX3 (5, 10 or 20 μg). (**B**) Survival curves of mice co-treated by PTX3 (20 μg) and CPS2 extraction (5, 25 or 25 μg). (**C**) The levels of IL-6 in the bloods of mice co-treated by PTX3 (20 μg) and CPS2 extractions (5, 25 or 50 μg). The Log-rank (Mantel–-Cox) test was used to analyze the survival curve in the animal experiments. A two-tailed unpaired *t* test was used for the rest of the experiments. For all tests, a *p* value < 0.05 was considered statistically significant (** *p* < 0.01, *** *p* < 0.001), and all the data were shown as mean ± SEM.

All the data above suggested that PTX3 can elicit strong immune responses and facilitate bacterial clearance during the SS2 infection, suggesting that PTX3 protein can be used as an efficient therapeutic agent or a vaccine adjuvant. After the mice were challenged with SS2 strain HA9801, the survival rates were 50%, 70%, and 10% for mice injected intravenously with 5, 10, and 20 μg exogenous PTX3, respectively; the survival rate was 25% for the mice infected by strain HA9801 alone (Figure 5A). Unfortunately, the injection of a larger dose (20 μg) of PTX3 protein did not have a higher survival rate compared to the doses of 5μg and 10 μg in the mouse systemic infection model, suggesting that the overdose of PTX3 may trigger an excessive inflammatory response, which will pose a great threat to the lives of infected mice. It should be noted that PTX3 injection alone at the dose of 20 μg could not cause death in mice (Figure 5B), implying that the excessive inflammatory response was caused by PTX3 and SS2 strain HA9801 coordinately. Given that CPS2 was required for triggering the host’s inflammatory response during SS2 infection, we speculated that the injections of CPS2 extraction at different doses may cause the death of mice co-treated with 20 μg PTX3 at different rates. Indeed, with the increase in CPS2 doses, the survival rates of mice co-treated with 20 μg PTX3 significantly declined from 100% for the 5 μg CPS2 dose to 40% for the 25 μg CPS2 dose (Figure 5B). This observation, coupled with the results suggesting that the IL-6 releases significantly increased in a dose-dependent manner for CPS2 supplement to a similar level as that with HA9801 infection under the co-treatment with 20 μg PTX3 (Figure 5C), verified that an excessive inflammatory response triggered by CPS2 and PTX3 caused mice deaths. The above data indicated that PTX3, when used as a therapeutic agent or vaccine adjuvant, must be evaluated strictly to ensure safety at an appropriate dose. All our findings suggested that *S. suis* surface CPS2 and host PTX3 proteins coordinately regulate the host’s inflammatory response during the infection of SS2.

## 4. Discussion

Because of its high pathogenicity, SS2 has become a significant zoonotic pathogen, causing significant economic losses in pig production and posing a great threat to human health. The current high morbidity and mortality rates of SS2, combined with the lack of an effective and safe vaccine formula, has resulted in the long-term use of antibiotics; excessive use of antibiotics has provoked the emergence of antimicrobial-resistant (AMR) *S. suis* isolates [34]. Therefore, effective vaccines and alternative therapeutic agents are urgently needed. Here, we explored the role of PTX3 as an important regulator of hosts’ innate immune resistance during the *S. suis* infection, and discovered that PTX3 facilitates bacterial clearance by enhancing the host’s inflammatory response.

The mouse air pouch is an ideal model for avoiding interference from other regulatory pathways within the blood stream, while also providing a visual and effective platform for evaluating the levels of inflammatory response during the *S. suis* infection. It greatly simplifies future research into the biological functions of the PTX3 protein in both this study and future studies. Exogenous PTX3 protein supplementation increased the levels of inflammatory cells and cytokines (IL-6) in the mouse air pouch model infected by SS2 strain HA9801, verifying the pivotal role of PTX3 as a mediator of the innate immune response during SS2 infection. Given the relative abundance of exudate compositions, it is difficult to precisely differentiate between all kinds of inflammatory cells from the samples. To minimize the potential errors caused by omissions in the identification of different types of inflammatory cells, we accounted for all of the cells in the exudate, verifying that neutrophils and monocytes are the main cellular components of the exudate by a microscopic examination. It should be noted that the presence of PTX3 alone did not trigger the inflammatory response, implying that its amplifying effect is dependent on bacterial component coordination. Indeed, subsequent research using the mouse air pouch model confirmed that the SS2 surface CPS2 is required for the PTX3 protein to modulate the host inflammatory response.

PTX3 protein contributes to the recruitment of a large amount of inflammatory cells (including neutrophils and other phagocytes) to infected tissues by amplifying the inflammatory response [20]. Meanwhile, PTX3 facilitates pathogen recognition by phagocytes and enhances their phagocytic vitality [19], regulating the clearance of invasive pathogens. In this study, we discovered that PTX3 facilitates the phagocytic ability of Ana-1 macrophages toward strain HA9801 in a dose-dependent manner (Figure 3) and significantly reduces the bacterial load of the infected organs in the mouse model (Figure 4). These results are consistent with previous research indicating that PTX3 plays a critical role in bacterial [14] and fungal infection defense [16], as well as in regulating the extent of tissue damage caused by non-infectious agents [35]. These findings proposed that PTX3 is required for host resistance to *S. suis* infection, emphasizing PTX3’s roles in triggering hosts’ innate immune responses and resistance to selected pathogens, and broadening its own spectrum of activity in infectious diseases.

Immunotherapy for pathogenic microorganism infections, as we all know, involves the use of vaccines or immunomodulatory agents, which stimulate the immune system to fight pathogens. Immunotherapy aims to boost the body’s natural defense mechanisms against pathogen infections, while also lowering the risk of severe or recurring infections. A heat-killed form of a non-pathogenic microbe––for example, Caulobacter crescents (HKCC)—could activate innate immunity and limit MTB infection [36]. Similarly, in the acute respiratory distress syndrome caused by COVID-19, specialized pro-resolving mediators (SPMs) can enhance macrophage polarization, activate the immune response and control acute inflammation in the body [37]. As a result, we hypothesized that PTX3 could be a novel immunomodulatory agent used in the prevention and treatment of streptococcal infections caused by SS2.

The administration of 10 μg exogenous PTX3 significantly improved the survival rate of mice challenged with *S. suis* strain HA9801, verifying the potential role of PTX3 as an effective therapeutic agent. Unexpectedly, mice treated with 20 μg PTX3 showed a sharp decline in survival rate when infected with strain HA9801. It is reasonable to conclude that a PTX3 overdose may stimulate the body to produce a violent inflammatory response, endangering the lives of infected mice. It should be noted that this type of excessive response was elicited by PTX3 and SS2 strain HA9801 in concert, with the overuse of CPS2 extraction causing a significant increase in mortality when the mice were co-treated with PTX3 at a fixed dose (20 μg/mouse). Indeed, exaggerated inflammatory responses can contribute to tissue damage and immunopathology [38,39], resulting in terrifying inflammatory storms and even death. A similar observation has also been demonstrated in *Klebsiella pneumoniae*: the overexpression of PTX3 in mice given a low dose of bacteria promoted resistance to *K. pneumoniae* respiratory infection, while in mice given a high inoculum, overexpression of PTX3 was associated with faster lethality. These results suggests the importance of PTX3-dependent inflammation tuning in this infection [40].

## 5. Conclusions

In conclusion, this study provided evidence that PTX3 is an innate inhibitor of SS2 infection both in vitro and in vivo; this finding suggests that PTX3 could be a novel biological agent used for the prevention and treatment of streptococcosis caused by SS2. However, the recommended dose of PTX3 must be evaluated strictly to avoid triggering an excessive inflammatory response that can cause serious tissue injury and animal death.

## Data Availability

Data available on request due to privacy restrictions.

## Supplementary Materials

The following supporting information can be downloaded at: https://www.mdpi.com/article/10.3390/vetsci10030239/s1, Figure S1: PTX3 facilitated CPS2 to trigger the release of cytokine IL-8 during the infection of SS2 HA9801. CPS2 contributed to the inflammatory response during SS infection in the mouse systemic infection model (A) and mouse air pouch model (B). (C) The release of cytokine IL-8 was activated by the co-treatment of CPS2 (25 μg) and PTX3 protein (5, 10, or 20 μg) in the mouse air pouch model. The statistical significance was calculated by two-tailed unpaired Student’s *t*-tests (** *p* < 0.01; *** *p* < 0.001).
