# Supplementary material for: Host PTX3 Protein and Bacterial Capsule Coordinately Regulate the Inflammatory Response during Streptococcus suis Infection
Source: Vet Sci. 2023 Mar 22;10(3):239. doi: 10.3390/vetsci10030239 (PMC10059727; doi:10.3390/vetsci10030239)
Supplement: Supplementary file 1 [file vetsci-10-00239-s001.zip › vetsci-2226933-supplementary.pdf]

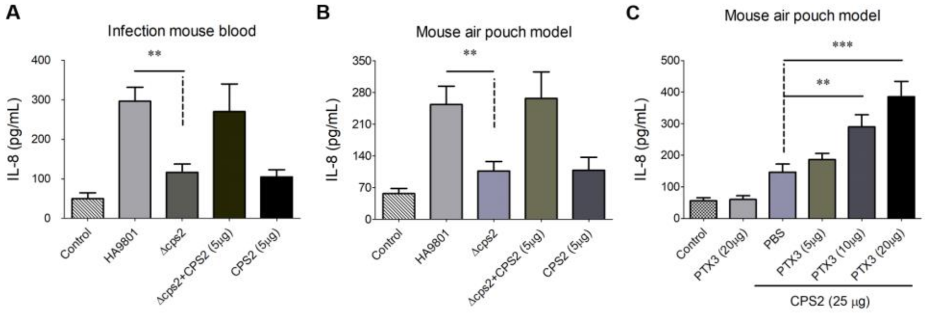

Figure S1. PTX3 facilitated CPS2 to trigger the release of cytokine IL-8 during the infection of SS2 HA9801. CPS2 contributed to the inflammatory response during SS infection in the mouse systemic infection model (A) and mouse air pouch model (B). (C) The release of cytokine IL-8 was activated by the co-treatment of CPS2 (25 μg) and PTX3 protein (5, 10, or 20 μg) in the mouse air pouch model. The statistical significance was calculated by two-tailed unpaired Student's *t*-tests (** $P < 0.01$; *** $P < 0.001$).
